# Botanicals in Dietary Supplements

**DOI:** 10.1155/2013/465313

**Published:** 2013-10-22

**Authors:** Weena Jiratchariyakul, Ludger Beerhues, Gail B. Mahady, Tanawan Kummalue, Molvibha Vongsakul

**Affiliations:** ^1^Department of Pharmacognosy, Faculty of Pharmacy, Mahidol University, 447 Sri Ayudhya Road, Rajathevi District, Bangkok 10400, Thailand; ^2^Institute of Pharmaceutical Biology, Technical University of Braunschweig, Mendelssohnstraße 1, 38106 Braunschweig, Germany; ^3^Clinical Pharmacognosy Laboratory, Department of Pharmacy Practice, PAHO/WHO Collaborating Centre for Traditional Medicine, College of Pharmacy, University of Illinois at Chicago, 833 S. Wood Street, MC 886, Rm 122, Chicago, USA; ^4^Department of Clinical Pathology, Faculty of Medicine, Siriraj Hospital, Mahidol University, 2 Prannok Road, Bangkoknoi, Bangkok 10700, Thailand; ^5^Department of Microbiology, Faculty of Science, Mahidol University, Rama 6 Road, Rajathevi District, Bangkok 10400, Thailand

Botanicals are accepted worldwide as medicinal agents and nutraceuticals. Extensive scientific investigations have been performed over the past 200 years which have resulted in the evolution of botanical utilization. This special issue highlights some of this research in 7 review articles and in 24 original research articles.

*Review Articles.* W. Jiratchariyakul and G. B. Mahady wrote about the botanical status and popular herbs in EU, US, and Thailand. M. Miroddi et al. updated the market and the regulatory of botanical products in EU and US. Traditional Chinese and Indian Medicines (TCM and TIM) play an important role in Asian countries. Z. Wang et al. presented and discussed the role of TCM in the treatment of epidemic type II diabetes mellitus. M. M. Pandey et al. reported the use of TIM as a nutritional supplement in malnutrition. Y. Kamisah et al. presented the chemoprevention and antioxidation of *Parkia speciosa*. A. P. Bartolome et al. reviewed laboratory evidence of *Bidens pilosa*. T.-P. Huynh et al. discussed the promising botanical compounds for prevention and treatment of eye diseases.

*Research Articles*. New biological and pharmacological activities of botanicals are reported. They included the enhancement of learning, memory and antistress of *Acanthopanax trifoliatus* (P. Sithisarn et al.), the sedative effect of *Ziziphus mauritiana* (A. M. M. San et al.), the cardioprotective effect of *Phyllanthus emblica* (L. Chularojmontri et al.), the cytoprotection and antioxidative stress of *Citrus maxima* (L. Chularojmontri et al.), anti-influenza viral activity of *Momordica charantia* (V. Pongthanapisith et al.), antioxidation from *Nypa fruticans* (N. Prasad et al.), *Nigella glandulifera* (J. Zhao et al.), TIM (M. M. Pandey et al.), *Herba Cynomorii* (J. Chen et al.). The alleviation of metabolic disorder of *Citrus ichangensis* (X. Ding et al.), and anticancer activity of Vitex agnus-castus (S. Li et al.).

The mechanisms of anticancer action are deeply investigated with the botanical compounds, zerumbone from *Zingiber zerumbet* (N. M. Nadzri et al.), phenyl butenoid dimmer from *Zingiber cassumunar* (T. Anasamy et al.), and girinimbine from *Murraya koenigii* (S. Mohan et al.). Biochanin A, the major isoflavone from *Trifolium pratense*, prevented the bone loss in the ovariectomized rat (S.-J. Su et al.). Quercetin isolated from *Caesalpinia mimosoides* had neuroprotective effect (N. Tangsaengvit et al.) and inhibited eosinophile (M. K. Asano Sakai-Kashiwahara). C. Li et al. reported new anti-inflammatory triterpenoids from *Illicium difengpi*.

Besides the herbal activities, the cohort study of TCM, Si-Wu-Tang, in postpartum women was performed to evaluate the health benefits. The effectiveness of the modern herbal drug was carried out using the double-blind randomized controlled clinical trial, as shown under the title “Antiherpetic effects of *Gynura procumbens*” (S. Jarikasem et al.).

The research on the quality assessment of the botanicals was also presented in this issue. It included the HPLC analysis of *Moringa oleifera* (B. Vongsak et al.) and *Pueraria tuberosa* (S. Rastogi et al.). In addition, the arsenic accumulation in *Zingiberaceous rhizomes* was reported (C. Ubonnuch et al.).

*Weena Jiratchariyakul* *Weena Jiratchariyakul* *Ludger Beerhues* *Ludger Beerhues* *Gail B. Mahady* *Gail B. Mahady* *Tanawan Kummalue* *Tanawan Kummalue* *Molvibha Vongsakul* *Molvibha Vongsakul*

